# Effects of trauma‐informed approaches in schools: A systematic review

**DOI:** 10.1002/cl2.1018

**Published:** 2019-07-17

**Authors:** Brandy R. Maynard, Anne Farina, Nathaniel A. Dell, Michael S. Kelly

**Affiliations:** ^1^ School of Social Work Saint Louis University St. Louis Missouri; ^2^ Department of Social Work Seattle University Seattle Washington; ^3^ School of Social Work Loyola University Water Tower Campus Chicago Illinois

## 1 PLAIN LANGUAGE SUMMARY

### 1.1 The review in brief

Despite growing support and increased rate of which trauma‐informed approaches are being promoted and implemented in schools, evidence to support this approach is lacking.

### 1.2 What is this review about?

Exposure to different types of trauma have been associated with varying types and complexity of adverse outcomes, including adverse effects on cognitive functioning, attention, memory, academic performance, and school‐related behaviors. Given the growing research on trauma and increased knowledge about the prevalence, consequences and costs associated with trauma, there have been increased efforts at the local, state and federal levels to make systems “trauma‐informed” (Lang, Campbell, & Vanerploeg, 2015). While the intent of creating trauma‐informed approaches in schools is a noble one, relatively little is known about the benefits, costs, and how trauma‐informed approaches are being defined and evaluated (Berliner & Kolko, 2016). Adopting a trauma‐informed approach in a complex system such as a school building or district is a time consuming and potentially costly endeavor and thus it is important to assess the effects of this approach to inform policy and practice.

This aim of this review was to assess trauma‐informed approaches in schools on trauma symptoms/mental health, academic performance, behavior, and socioemotional functioning. Trauma‐informed approaches include programs, organizations, or systems that realize the impact of trauma, recognize the symptoms of trauma, respond by integrating knowledge about trauma policies and practices, and seeks to reduce retraumatization. At least two of the three key elements of a trauma‐informed approach must have been present: Workforce development, trauma‐focused services, and organizational environment and practices, which differ from trauma‐specific interventions designed to treat or otherwise address the impact/symptoms of trauma and facilitate healing.

**What is the aim of this review?**

This Campbell systematic review sought to examine the effects trauma‐informed schools on trauma symptoms/mental health, academic performance, behavior, and socioemotional functioning. Although we conducted a comprehensive search to find studies testing trauma‐informed approaches in schools, no studies met the inclusion criteria.

### 1.3 What are the main findings of this review?

No studies met criteria for this review, indicating that there is a lack of evidence of trauma‐informed approaches in schools.

### 1.4 What do the findings of this review mean?

Despite widespread support and growing adoption of trauma‐informed approaches in schools across the globe, we found no studies to provide good evidence to suggest that this approach is effective in achieving the stated goals. Given the degree to which trauma‐informed approaches are being adopted in schools across the US and other countries, it is important that the effects of these programs be assessed.

### 1.5 How up‐to‐date is this review?

The review authors searched for studies June through September, 2017.

## 2 EXECUTIVE SUMMARY/ABSTRACT

### 2.1 Background

Exposure to different types of trauma have been associated with varying types and complexity of adverse outcomes, including adverse effects on cognitive functioning, attention, memory, academic performance, and school‐related behaviors. Given the growing research on trauma and increased knowledge about the prevalence, consequences and costs associated with trauma, there have been increased efforts at the local, state and federal levels to make systems “trauma‐informed” (Lang et al., 2015). Indeed, federal legislation has been proposed to advance trauma‐informed practice, with approximately 49 bills introduced between 1973 and 2015 that explicitly mentioned trauma‐informed practice, with more than half introduced in 2015 alone (Purtle & Lewis, 2017). The promotion and provision of trauma‐informed approaches in school settings in particular is growing at a rapid rate across the United States. At least 17 states have implemented trauma‐informed approaches at the school, district, and even state‐wide levels (Overstreet & Chafouleas, 2016). This rapid increase in the growth of trauma‐informed approaches in schools has been fueled by a number of local, state, and federal initiatives and increasing support by education related organizations. While the intent of creating trauma‐informed approaches in schools is a noble one, relatively little is known about the benefits, costs, and how trauma‐informed approaches are being defined and evaluated (Berliner & Kolko, 2016). Adopting a trauma‐informed approach in a complex system such as a school building or district is a time consuming and potentially costly endeavor, and there is potential for harm; therefore, it is important to assess the effects of this approach to inform policy and practice.

### 2.2 Objectives

The purpose of this review was to identify, describe and synthesize the evidence of effects of trauma‐informed approaches in schools to provide guidance for policymakers and educators and to identify important gaps in the evidence base.

### 2.3 Search methods

We conducted a search for published and unpublished studies using a comprehensive search that included nine electronic databases and searches of various research registers, gray literature sources, reference lists of prior reviews and relevant studies, and contacts with authors and researchers in the field of trauma and school‐based intervention research.

### 2.4 Selection criteria

Criteria for inclusion in the review included:

1. Must have used a randomized or quasi‐experimental study design in which participants who received an intervention were compared with a wait‐list, no treatment, treatment‐as‐usual or an alternative treatment comparison group.
2. Studies must have been conducted in a school setting serving PreK‐12 (or equivalent) students.
3. Studies must have assessed effects of a trauma‐informed approach, defined as a program, organization, or system that realizes the impact of trauma, recognizes the symptoms of trauma, responds by integrating knowledge about trauma policies and practices, and seeks to reduce retraumatization. At last two of the three key elements of a trauma‐informed approach must have been present: Workforce development, trauma‐focused services, and organizational environment and practices (Hanson & Lang, 2016). This approach is distinguished from trauma‐specific interventions, which are specific interventions designed to treat or otherwise address the impact/symptoms of trauma and facilitate healing.
4. Studies must have measured a student‐level outcome related to trauma symptoms/mental health, academic performance, behavior, or socioemotional functioning.
5. We did not limit studies based on publication status, geographical location or language. We searched for studies that had been published in the last 10 years, as this is a relatively recent movement

### 2.5 Data collection and analysis

One reviewer searched all sources and uploaded all potentially relevant citations to Covidence, a systematic review software, for further screening by two reviewers. Two reviewers then independently screened each of the full‐text reports for eligibility using a screening instrument. Disagreements related to eligibility were discussed and resolved between the two reviewers. Data extraction and analysis was not possible due to no studies having met criteria for inclusion in the review.

### 2.6 Results

A total of 9,102 references from all searches were imported to Covidence for screening. After removal of 1,929 duplicates, 7,173 titles/abstracts were screened, and 7,106 studies were excluded. The remaining 67 studies were assessed for full‐text eligibility by two independent reviewers. All 67 studies were excluded: 49 were neither an randomized controlled trial (RCT) nor quasi‐experimental design (QED); 12 did not examine effects of a trauma‐informed approach; 5 examined only one aspect of a trauma‐informed approach (only workforce OR organizational OR practice changes); one was not a school‐based intervention. Some studies may have been excluded for multiple reasons; however, only the first (primary) reason for exclusion was recorded. See Figure 1 for flowchart of the search and selection process. A full list of excluded studies can be found in References to Excluded Studies.

No studies met criteria for inclusion in this review.

### 2.7 Authors' conclusions

Trauma‐informed approaches are being promoted and used across child‐serving systems, and the number of states and school districts adopting trauma‐informed approaches in schools is growing rapidly (Overstreet & Chafouleas, 2016). While the premise of a trauma‐informed schools approach is a noble one, it is unclear as to whether the promise of this framework is actually delivering the types of systemic and programmatic changes intended, and if those changes are resulting in the outcomes the proponents of a trauma‐informed approach in schools hoped for. The purpose of this systematic review was to find, describe, evaluate, and synthesize effects of trauma‐informed approaches in schools to inform policy and practice. While there are a number of publications that describe trauma‐informed approaches, advocate for the need for trauma‐informed approaches, and discuss the potential benefits of adopting such an approach in schools, we found no rigorous evaluations through our extensive search process.

From this review, it seems like the most prudent action for school leaders, policymakers, and school mental health professionals to do would be to proceed with caution in their embrace of a trauma‐informed approach as an overarching framework and begin evaluating these programs. We simply do not have the evidence (yet) to know if this approach works, and indeed, we also do not know if implementing trauma‐informed approaches in schools could have unintended negative consequences for traumatized youth and school communities. We also do not have evidence of other potential costs in implementing this approach in schools, whether they be financial, academic, or other opportunity costs, and whether benefits outweigh the costs of implementing and maintaining this approach in schools.

That said, calling for caution in adopting a trauma‐informed approach in schools does not preclude schools from continuing to implement evidence‐informed programs that target trauma symptoms in youth, or that they should simply wait for the research to provide unequivocal answers. We do encourage healthy skepticism and evaluation by the schools who are adopting a trauma‐informed approach and clear descriptions of what schools are doing. Currently, despite several theoretical and guidance documents, it is not clear exactly what schools are doing when they say they are using a trauma‐informed approach. Not only do we need more research on the effects, but descriptive and qualitative research on what is actually being implemented would be a welcome addition to the empirical literature in this area. We suspect that schools may be calling what they are doing a trauma‐informed approach, but what is actually being done from school to school or district to district may vary quite widely in the practice and implementation of this approach. Clearly, rigorous research is needed in assessing the effects of using a trauma‐informed approach in schools and we encourage rigorously designed studies in this area. Evaluating complex interventions such as this is not easy and requires resources. Drawing from research on multi‐tiered approaches in schools could help inform research approaches to assess the effects (and costs) of trauma‐informed approaches in schools.

## 3 BACKGROUND

### 3.1 The problem, condition, or issue

Childhood trauma has been receiving increased attention and it is increasingly being recognized as a significant public health concern (Lang et al., 2015). Trauma exposure involves “actual or threatened death, serious injury, or sexual violence” that is either directly experienced or witnessed, learning that any traumatic experiences have happened to a loved one, or having repeated exposure to details of traumatic events (American Psychiatric Association, 2013, p. 271). Prevalence estimates of trauma experienced in childhood or adolescence vary by type of traumatic event (e.g., physical abuse, neglect, sexual abuse, witnessing violence, natural disasters) and how and when the traumatic experience is measured, but can range between 4% and 71% (Finkelhor, Turner, Shattuck, & Hamby, 2015; McLaughlin et al., 2013; Saunders & Adams, 2014). Prevalence and also vary by sociopolitical context as some countries are affected by war and have much higher levels of trauma (Bosqui, Marshoud, & Shannon, 2017).

Exposure to traumatic events can disrupt brain development and can have immediate and lifelong adverse effects on social, emotional, and physical wellbeing, including deficits in executive functioning, developmental delays, behavioral health problems, difficulty regulating emotions and behavior, academic performance and IQ, school behavior problems, delinquency, substance abuse, and mental health and psychiatric disorders (Anda et al., 2006; Delaney‐Black et al., 2002; DePrince, Weinzierl, & Combs, 2009; Flannery, Wester, & Singer, 2004; Lang et al., 2015; Lansford et al., 2002). In a systematic review specifically examining school‐related outcomes of traumatic event exposure, Perfect, Turley, Carlson, Yohannan, and Gilles (2016) identified 44 studies that examined cognitive functioning, 34 that examined academic functioning, and 24 that examined social‐emotional‐behavioral functioning. Their findings suggest that youth who have experienced trauma are at significant risk for impairments across various cognitive functions, including IQ, memory, attention and language/verbal ability; poorer academic performance and school‐related behaviors such as discipline, dropout and attendance; and higher rates of behavioral problems and internalizing symptoms.

Exposure to different types of trauma have been associated with varying types and complexity of adverse outcomes. Kira, Lewandowski, Somers, Yoon, and Chiodo (2012) study of African American and Iraqi refugee youth found that different types of trauma differentially impact different components of cognitive functioning, including perceptual reasoning, working memory, processing speed and verbal comprehension. In another study examining effects of different types of trauma, exposure to violence was found to be associated with depression, separation anxiety, and conduct problems, whereas exposure to noninterpersonal traumatic events was associated with phobic anxiety (Briggs‐Gowan et al., 2010). Moreover, there is some evidence that the effects of trauma are cumulative, thus youth who experience a greater number of traumatic events are more at risk for adverse outcomes and more complex symptoms through adulthood (Chartier, Walker, & Naimark, 2010; Cloitre et al., 2009; Hodges et al., 2013). Duke, Pettingell, McMorris, and Borowsky (2010) analyzed data from respondents to the 2007 Minnesota Student Survey (n = 136,549), and identified “a significant positive relationship between each adverse event and delinquent behaviors for girls and boys,” (p. e782). The effects of cumulative trauma also go beyond frequency, as the type, severity, and duration of trauma has been shown to be important (e.g., childhood sexual abuse has been found to have a stronger association with negative adult outcomes than other forms of abuse and neglect; Bebbington et al., 2004; Bosqui et al., 2014).

While exposure to traumatic or potentially traumatic experiences are associated with a range of short and long‐term outcomes, there are multiple pathways through which trauma can impact various domains across the life course. Moreover, not all youth will experience the same traumatic events in the same way and not all youth will develop symptoms following a traumatic experience (Layne et al., 2009).

Given the growing research on trauma and increased knowledge about the prevalence, consequences and costs associated with trauma, there have been increased efforts at the local, state, and federal levels to make systems “trauma‐informed” (Lang et al., 2015). In an effort to examine the extent to which federal legislation has been proposed to advance trauma‐informed practice, Purtle and Lewis (2017) conducted a policy mapping study of federal legislative proposals from 1973 to 2015 that explicitly mentioned trauma‐informed practice. The authors identified 49 bills introduced, beginning in December 2009 with the Domestic Minor Sex Trafficking Deterrence and Victims Support Act, and observed a dramatic increase over time with 28 bills being introduced in 2015 alone. Altogether, the 49 bills contained 71 sections that included trauma‐informed language, with the highest proportion of those specifically targeting youth in primary and secondary schools (16 sections, 22.5%). For example, the United States Congress established the National Child Traumatic Stress Network (NCTSN) in 2000 through a congressional initiative that is funded by the Substance Abuse and Mental Health Services Administration (SAMHSA). The National Child Traumatic Stress Network (n.d.a) is a growing network of providers, researchers, and families with a broad mission to improve care and access to services for traumatized children, their families, and communities. The NCTSN offers training, support, and resources aimed at treatment, intervention development, program evaluation, systems change, and the integration of trauma‐informed and evidence‐based practices in all child‐serving systems. The Administration for Children and Families, Center for Medicare and Medicaid Services, the Department of Justice, and the Department of Education have also recognized the impact of child trauma on youth wellbeing and development and are launching initiatives and implementing policies designed to promote the use and expansion of trauma‐informed systems and programs across child‐serving organizations and agencies (Lang et al., 2015; Substance Abuse and Mental Health Services Administration, 2014).

The promotion and provision of trauma‐informed approaches in school settings is growing at a rapid rate across the United States. At least 17 states have been identified in which trauma‐informed approaches have been implemented at the school, district, and even state‐wide levels (Overstreet & Chafouleas, 2016). This rapid increase in the growth of trauma‐informed approaches in schools has been fueled by a number of local, state, and federal initiatives and increasing support by education related organizations. For example, there are explicit provisions for trauma‐informed practices in the Every Student Succeeds Act (Every Student Succeeds Act, 2015), the legislation that replaced No Child Left Behind, including training of school personnel in understanding when and how to refer students affected by trauma, and grant programs that provide funding to support services that are based on trauma‐informed practices that are evidence‐based (section 4108). The promotion of trauma‐informed schools is also supported by the National Education Association and state‐level agencies have been spearheading efforts to develop guidelines and implement change within and across school systems.

Globally, there is understanding of the impact of trauma on children and the consequences for the school environment. For example, multiple systems in Australia (child welfare, disability support, human services, mental health, legal context) have identified the need for using a trauma‐informed lens in practice. The Queensland, Australia educational system was particularly interested in ensuring that the educational and mental health needs were met for children living in out‐of‐home care and children with complex trauma histories and therefore looked to a trauma‐informed framework as a possible intervention to meet these needs. Prompted by limited success in meeting the challenges schools in Australia face with students with serious behavior concerns, the Queensland University of Technology and the Department of Education in Queensland conducted a study to explore the understanding and need for trauma‐aware schooling and identified the need for comprehensive training and support for school personnel (Howard, 2018).

In the United Kingdom, to improve the connection between mental health services and the education system, the Department for Education and National Health Service have conducted multiple surveys and pilot projects in attempts to provide greater school‐wide approaches to promote mental health and wellbeing for children. They have identified trauma, attachment, and post‐traumatic stress as key areas where schools need guidance (Department of Health & Department for Education, 2017). In response to the publication of the Department of Education and National Health Service, organizations such as the Center for Mental Health responded, urging the Government to put forth the adequate resources to implement whole school approaches and not only invest in trauma‐focused interventions, but to also address the underlying causes that contribute to children's mental health (Hughes, n.d.).

In areas of the world where there is conflict, emergency, and crisis, both children and adults see schools as places of refuge, learning, and paths to better futures. International organizations such as Save the Children have identified education as priority for children and they urge both national governments and humanitarian actors to ensure that children have access to schooling and to ensure schools are providing the appropriate mental health support (Save the Children, 2015). The United Nations Girls' Education Initiative in the East Asia Pacific Region, which aims to ensure that both boys and girls receive primary and secondary education, have identified the need for additional support services for those who have experienced trauma (Clark & Sawyer, 2014). With limited resources and surrounding conflict, the knowledge of what environment would best support children's learning may be understood, but the lack of resources may prevent schools from implementing trauma‐informed approaches.

### 3.2 The intervention

Trauma‐informed approaches are being promoted and used across child‐serving systems and constitute a relatively new approach to trauma care for children and youth being served within the child welfare, juvenile justice, mental health, and education systems. While trauma‐specific interventions, such as Trauma‐Focused Cognitive Behavioral Therapy, are well known and widely used to treat trauma‐related symptoms and disorders in both adults and children, trauma‐informed approaches to care are distinct from trauma‐specific interventions. However, what is essential to a “trauma‐informed approach” has not always been clearly operationalized, and the approach and variations of the approach have been referred to in varying ways, for example, “trauma‐informed care,” “trauma‐sensitive,” “trauma‐informed system” (Hanson & Lang, 2016). To date, there is no consensus on the use of these terms, which makes efforts to both implement and study trauma‐informed approaches to care challenging.

**Figure 1:**
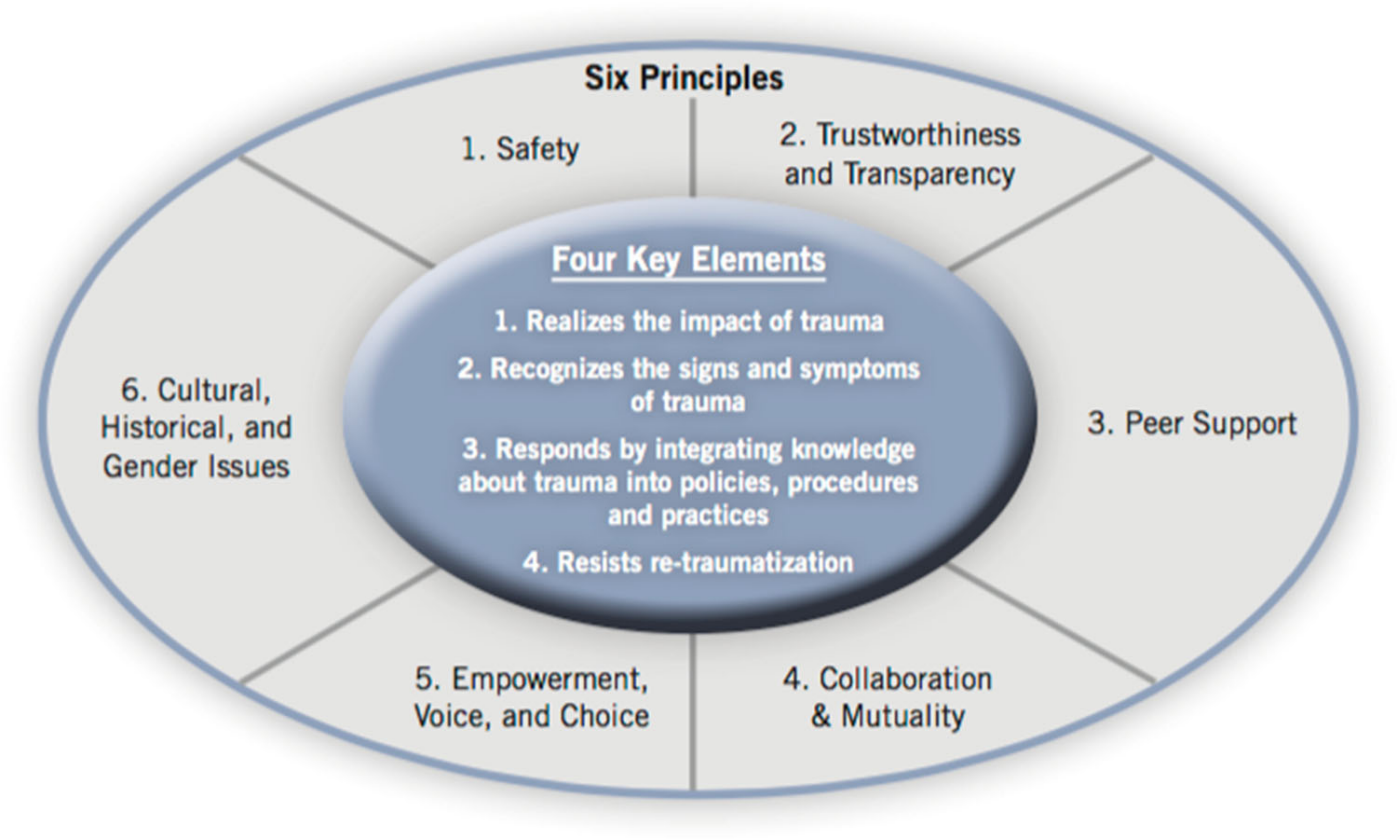
SAMHSA's trauma‐informed approach (Lang et al., 2015). SAMHSA, Substance Abuse and Mental Health Services Administration

While there is much confusion, overlap and misuse of the various terminologies in this rather nascent area of practice and research, we are drawing from SAMHSA and the NCTSN to define trauma‐informed approaches for the purpose of this review. Substance Abuse and Mental Health Services Administration (2014) defines trauma‐informed approaches (which the agency uses interchangeably with “trauma‐informed care”) as incorporating “key trauma principles into the organizational culture” of the program, agency, or system (p. 9). A trauma‐informed approach is thus more akin to a multi‐tiered framework such as School‐Wide Positive Behavioral Supports (Chafouleas, Johnson, Overstreet, & Santos, 2016), and is based on incorporating four key assumptions and six key principles, generalizable to any setting, that are infused across all levels of an organization rather than implementing a prescribed set of practices or interventions (Substance Abuse and Mental Health Services Administration, 2014; see Figure 1).

A trauma‐informed program, organization, or system is one that (Substance Abuse and Mental Health Services Administration, 2014, p. 9):

1. Realizes the widespread impact of trauma and understands potential paths for recovery;
2. Recognizes the signs and symptoms of trauma in clients, families, staff, and others involved with the system;
3. Responds by fully integrating knowledge about trauma into policies, procedures, and practices;
4. Seeks to actively resist retraumatization of both persons served and staff.

The six key principles of a trauma‐informed approach include safety; trustworthiness and transparency; peer support; collaboration and mutuality; empowerment, voice and choice; and cultural, historical, and gender issues (Substance Abuse and Mental Health Services Administration, 2014).

The National Child Traumatic Stress Network (n.d.b) adapts the model outlined by SAMHSA to a context specific to youth and families, and describes their model as a “trauma‐informed child‐ and family‐service system,” in which:

all parties involved recognize and respond to the impact of traumatic stress on those who have contact with the system, including children, caregivers, and service providers. Programs and agencies within such a system infuse and sustain trauma awareness, knowledge, and skills into their organizational cultures, practices, and policies. They act in collaboration with all those who are involved with the child, using the best available science, to facilitate and support the recovery and resiliency of the child and family.

In essence, a trauma‐informed approach is not a standalone intervention that can be delivered in isolation, but rather a framework to guide systems. A trauma‐informed approach can include trauma‐specific interventions, but trauma‐specific interventions alone are not seen as sufficient for achieving optimal outcomes or to influence service systems (Substance Abuse and Mental Health Services Administration, 2014). Hanson and Lang (2016) identify three core domains essential to trauma‐informed care that they derived from analyzing definitions and components across several organizations and authors, including SAMHSA and NTCSN: (a) Workforce/professional development (PD), (b) organizational changes, and (c) practice changes.

**Examples of trauma‐informed approaches implemented in schools**

As described above, trauma‐informed approaches are complex interventions and involve a number of components at various levels, thus providing some examples of such programs can be helpful in elucidating this complexity. One example of a trauma‐informed approach is the Healthy Environments and Response to Trauma in Schools (HEARTS) program that was implemented in elementary schools (K‐8th grade) in the San Francisco Unified School District. HEARTS is a whole‐school program developed by the University of California, San Francisco to promote school success for students who have been impacted by trauma (Dorado, Martinez, McArthur, & Leibovitz, 2016). This whole‐school approach used the response to intervention three‐tier framework of universal, selected and targeted interventions and included supports at the system, adult (teacher/staff) and student levels at each tier. Activities involved changes in school policies and school‐wide practices; training, PD, and consultation for all school staff around trauma‐sensitive practices and stress, burnout and secondary trauma; and use of evidence‐informed universal, secondary and targeted trauma‐informed interventions. TRUST in Schools: Trauma, Understanding & Sensitive Teaching is an example of a trauma‐informed school approach being piloted in Australia (Harris, n.d.). This program focuses on recognizing the impact of trauma on children across the whole school, a school executive that promotes trauma‐sensitive policies and procedures in the school, supporting school staff in implementing sensitive practices and engaging families, carers, and school communities in understanding the need for a trauma‐sensitive whole school approach.

Trauma‐informed approaches are also used at the preschool level. A multiorganization collaboration implemented a trauma‐informed approach in head start programs in the Appalachian region of the US (Shamblin, Graham, & Bianco, 2016). This comprehensive, three‐tier model involved the use of trained consultants in the classroom to provide three tiers of services. The first tier was universal consultation, intended to build teacher capacity to deliver an evidence‐based social‐emotional curriculum (Second Steps or Incredible Years) to children and help teachers understand trauma‐informed principles through training and mentoring of teachers. The second tier involved targeted consultation of teachers to develop behavior plans and specific strategies to address challenging behaviors of individual children in the classroom which take into account the child's trauma experience. The third tier included the provision of intensive services wherein the consultant provided on‐site mental health assessment and treatment to children and their families. For children who had experienced trauma, the consultant provided Trauma‐Focused Cognitive Behavior Therapy and/or Parent–Child Interaction Therapy. In addition, the collaboration provided workforce development training to preschool teacher and other child service providers at various times during the year.

While schools may be implementing trauma‐informed approaches, it is unclear to what extent or how much variation there is in what schools are implementing or how much emphasis they are putting on various components (e.g., workforce development versus organizational change versus practice changes) and whether schools may implement trauma‐informed approaches differently based on the characteristics of their students, neighborhood, country or other contextual factors.

### 3.3 How the intervention might work

One out of every four children attending school has been exposed to a traumatic event, which can impact school performance, impair learning, and cause physical and emotional distress (NCTSN, 2008). Moreover, the majority of youth who have experienced trauma do not receive services, and those who are exposed to a potentially traumatic event do not necessarily need an intervention (Layne et al., 2009). Due to the relatively high rates of youth exposed to traumatic events and the negative impacts of those traumatic experiences on academic achievement and life course outcomes, schools represent a natural system in which to help prevent and reduce the adverse effects of trauma and more effectively engage students in the learning process (Chafouleas et al., 2016). Trauma‐informed schools adopt the trauma‐informed approach to “create educational environments that are response to the needs of trauma‐exposed youth through the implementation of effective practices and system‐change strategies” (Overstreet & Chafouleas, 2016, p. 1). A trauma‐informed approach in schools is designed to create a systematic model for schools to decrease the impact of trauma on students (Wiest‐Stevenson, & Lee, 2016) and more appropriately address academic, behavioral and socioemotional problems by recognizing and responding to student behavior from a trauma‐informed perspective. This is done through a multilevel approach intended to improve the school environment through implementing trauma‐informed policies and procedures; increase the ability of school staff to recognize and more effectively respond to students through PD; and prevent, mitigate and reduce trauma‐associated symptoms through evidence‐informed practices, leading to improved student academic, behavioral, and socioemotional outcomes.

As described above, a trauma‐informed approach involves strategies implemented at various levels in the school and includes workforce/PD, organizational change, and practice change using evidence‐informed practices (Hanson & Lang, 2016).

#### 3.3.1 Workforce/PD

The workforce and PD component is intended to increase staff knowledge about the prevalence and effects of trauma and associated cognitive, behavioral, and socioemotional effects of trauma. In addition, PD is intended to increase staff's ability to recognize signs and symptoms of trauma and improve skills in appropriately responding to students exhibiting trauma symptoms so that staff can more effectively address student behavior and make appropriate referrals for more targeted services. For example, typical strategies school staff use when addressing disruptive behavior that focus on consequences for misbehavior can exacerbate problems with trauma victims and miss an opportunity to more effectively intervene. By being trained to use a trauma‐informed lens, school staff can proactively prevent and deescalate problematic behaviors that would typically disrupt the classroom and student learning, improving the learning environment for the entire class, and positively impact students' behavior, socioemotional and academic outcomes (Lang et al., 2015). In addition, workforce development initiatives may also promote the recognition of and skills to cope with secondary stress and prevent burnout, which may occur in providers serving traumatized populations.

#### 3.3.2 Organizational environment and practices

Schools may implement any of the following policies and procedures to realize organizational changes that maximize learning and reduce incidences of both traumatization and retraumatization. Changes may include: Modifying disciplinary practices, which contextualize the notion of “accountability” within an understanding of common reactions to trauma, minimize disruption in education, and model respectful relationships; establishing protocols for communication among caregivers, the school, and community agencies; modifications to the school's physical environment to promote safety; and, fostering partnerships with and linkages to community health and mental health resources (Cole et al., 2009).

#### 3.3.3 Trauma‐focused practices

The practice change component to trauma‐informed schools involves implementing screening and universal, selective and/or indicated intervention programs that incorporate knowledge about trauma and are evidence‐informed. Schools may directly provide screening and intervention services in the school or collaborate with other providers to either implement programs and services in the school or refer students for screening and services in the community. Ideally, trauma‐informed schools would provide screening and interventions at all levels either directly or indirectly; however, some schools may not have the resources to provide all levels of screening and intervention.

### 3.4 Why it is important to do the review

Although one could argue about the necessity or value of schools adopting a trauma‐informed approach, trauma‐informed approaches are being promoted and used across child‐serving systems, and the number of states and school districts adopting trauma‐informed approaches in schools is growing rapidly (Overstreet & Chafouleas, 2016). While the intent of creating trauma‐informed approaches in schools is a noble one, there is relatively little known about the benefits, costs, and how trauma‐informed approaches are being defined and evaluated (Berliner & Kolko, 2016). Indeed, it is unclear whether schools adopting a trauma‐informed approach (i.e., being a “trauma‐informed school”) are effective in reducing trauma symptoms or affecting behavioral or academic outcomes, as the proponents of the movement propose. Adopting a trauma‐informed approach in a complex system such as a school building or district is a time consuming and potentially costly endeavor and thus it is important to assess the effects of this approach to inform policy and practice. Also, from our cursory review of the literature, the description of the trauma‐informed approaches being implemented in schools vary in terms of the types of strategies used in each of the three areas and the relative emphasis on the three areas (policy/procedures, PD, and practices), thus it is important to conduct a systematic inventory and description of the trauma‐informed approaches implemented in schools to more fully understand how this approach is being utilized in schools, and whether variations in the components impact outcomes.

To date, we have not located any systematic reviews specifically examining effects of trauma‐informed approaches in schools. We have identified reviews that have examined trauma‐specific interventions for adolescents (e.g., Black, Woodworth, Tremblay, & Carpenter, 2012; Cary & McMillen, 2012) and those that examine school‐based interventions for specific trauma‐related disorders, such as PTSD (e.g., Rolfsnes & Idsoe, 2011); however, these reviews are examining effects of trauma‐specific interventions rather than trauma‐informed approaches. By virtue of these studies' primary research questions and inclusion criteria, the scope of these reviews were not designed to examine effects of a trauma‐informed approach in schools.

## 4 OBJECTIVES

This purpose of this review was to identify, describe and synthesize the evidence of effects of trauma‐informed approaches in schools to provide guidance for policymakers and educators and to identify important gaps in the evidence base.

Specifically, the research questions guiding this review include:

1. What evidence is available to examine the effectiveness of trauma‐informed practices in schools?
2. What are the study, intervention, and participant characteristics of studies that have rigorously evaluated the effects of trauma‐informed schools?
3. What are the components of trauma‐informed approaches being used in schools?
4. What are the effects of trauma‐informed schools on trauma symptoms, socioemotional outcomes, behavior, and academic outcomes?
5. Are there certain components of trauma‐informed approaches that are more effective than others?
6. What adverse outcomes are reported by authors?

## 5 METHODS

### 5.1 Criteria for considering studies for this review

A protocol for this review was published in the Campbell Collaboration Library (Maynard, Farina, & Dell, 2017).

#### 5.1.1 Types of studies

To be included in this review, studies must have used one of the following research designs: RCT or QED with a treatment and comparison group using a wait list control, no treatment, treatment‐as‐usual and alternative treatment control group; therefore, single group pre‐post test studies will be excluded. We excluded studies in which the comparison group received an intervention that would meet criteria as a trauma‐informed approach (defined below), but included studies in which comparison groups received an alternative treatment that did not meet that criteria (e.g., a comparison group receiving a standalone trauma intervention would be included). The type of comparison group used in each study was coded. Given the nascent nature of research in this area, we anticipate lower‐quality quasi‐experimental and experimental studies. Although higher quality designs provide higher quality evidence, we were interested in capturing the research that currently exists and describing the quality of that research to inform research development in this area. Therefore, we did not require that studies provide pretest data or make statistical adjustments; however, we planned to code study design and analysis elements and use these variables in sensitivity and moderator analyses if there were a sufficient number of studies.

#### 5.1.2 Types of participants

We included studies that examined effects of the intervention in a school setting serving students in preschool through 12th grades (or equivalent grade levels in other countries).

#### 5.1.3 Types of interventions

Substance Abuse and Mental Health Services Administration (2014) defines a trauma‐informed approach as a program, organization, or system that realizes the impact of trauma, recognizes the symptoms of trauma, responds by integrating knowledge about trauma policies and practices, and seeks to reduce retraumatization. Three key elements of a trauma‐informed approach include workforce development, trauma‐focused services, and organizational environment and practices (Hanson & Lang, 2016). SAMHSA distinguishes between a trauma‐informed approach from trauma‐specific interventions, the latter which are specific interventions designed to treat or otherwise address the impact/symptoms of trauma and facilitate healing. We intended to examine the effects of trauma‐informed approaches implemented in school settings, often referred to as trauma‐informed schools.

We anticipated that there would be wide variation in the implementation of the trauma‐informed approach used in schools and variability in the principles and practices adopted by schools. We believe that identifying and describing this variation will be a significant contribution to the literature as currently “trauma‐informed schools” is often discussed as if everyone agrees on what this means or that any effort to become a “trauma‐informed school” will be equally meaningful and effective.

Therefore, we wanted to be able to discern between studies examining trauma‐specific interventions and those that were attempting a more comprehensive trauma‐informed approach while not being overly limiting. Thus, for the purposes of this review, the intervention was considered a trauma‐informed school approach if at least two of the following three components were present:

1. Workforce/PD‐components of the program are designed to increase knowledge and awareness of school staff on the impact, signs and symptoms of trauma, including secondary traumatization. PD did not necessarily have to be provided to all school staff in a school, but there must be some staff development component as part of the program.
2. Organizational change‐may include school‐wide policies and procedures and/or strategies or practices intended to create a trauma‐informed environment integrating the key principles of the trauma‐informed approach.
3. Practice change and use of evidence‐informed trauma practices‐ the program must implement changes in practice behaviors across the school, including trauma‐specific screening, prevention and/or intervention services.

We planned to code each of the components and describe whether studies included all three components, and if not, which components were included.

#### 5.1.4 Types of outcome measures

*Primary Outcomes.* Studies must have measured at least one of the following student‐level outcomes:

- Trauma symptoms/mental health outcomes (e.g., anxiety, depression, post‐traumatic stress disorder)
- Academic performance (e.g., standardized achievement tests, measures of content mastery, reading, grades)
- Behavior (e.g., disciplinary referrals, aggression and other externalizing behaviors, time on task, compliance, attendance)
- Socioemotional (e.g., stress, engagement, social skills, self‐esteem, emotion regulation, grit)

Measurement of above outcomes may have been conducted using standardized or unstandardized instruments using self‐, parent‐, or teacher‐reported or researcher administered measures. To be included in the meta‐analysis, primary study authors must have reported enough information to calculate an effect size. If sufficient information to calculate an effect size was not provided, we planned to make every effort to contact primary study authors and request the necessary information.

##### 5.1.4.1 Secondary outcomes

We anticipated study authors may measure additional outcomes at different levels (individual, classroom, school) and planned to report teacher outcomes and outcomes related to implementation (e.g., satisfaction, fidelity) if reported. We were also interested in reporting of adverse outcomes. For all outcomes that do not fit into one of the primary outcome categories as noted above, we planned to code the outcomes and categorize them post hoc for descriptive purposes. If there were a sufficient number of studies reporting the same outcomes, we planned to extract effect size data and conduct a meta‐analysis.

#### 5.1.5 Duration of follow‐up

We planned to include measurement points at posttest and all follow‐up time points and synthesize outcomes across studies that reported similar follow‐up time points (i.e., up to 3 months, 3–6 months, 6–12 months, >12 months) if there were more than two studies that report sufficient data.

#### 5.1.6 Types of settings

We included studies of interventions conducted in a preschool through 12th grade (or equivalent) school setting.

#### 5.1.7 Other criteria

We did not limit studies based on publication status, geographical location, or language. We searched for studies that had been published in the last 10 years, as this is a relatively recent movement.

### 5.2 Search methods for identification of studies

We conducted a search for published and unpublished studies using a comprehensive search that included multiple electronic databases, research registers, gray literature sources, and reference lists of prior reviews and relevant studies, and contacts with authors and researchers in the field of trauma and school‐based intervention research.

#### 5.2.1 Electronic databases

a. Academic Search Complete
b. Database of Research on International Education
c. Education Source
d. ERIC
e. MEDLINE
f. ProQuest Dissertations and Theses
g. PsycINFO
h. Social Science Citation Index
i. CINAHL

#### 5.2.2 Search terms and keywords

We used combinations of terms related to the intervention, population, study design, and setting to search the electronic databases. Database‐specific strategies were explored for each database, including the use of truncation and database‐specific limiters and thesauri were consulted to employ more precise search strategies within each database. Below are examples of the types of terms we used. See Table 1 in Tables and Figures for the full search strategy for each database.

**Table 1:** Electronic database search strategy

Database	Search terms	Date searched
Academic Search Complete	KW ("trauma‐informed" OR "trauma‐sensitive" OR “trauma services” OR trauma OR PTSD OR “post‐traumatic stress disorder”) AND (“elementary school” OR “primary school” OR “high school” OR “secondary school” OR “middle school” OR kindergarten OR "pre‐kindergarten" OR child* OR youth OR adolescent OR school) AND (evaluation OR intervention OR treatment OR outcome OR program OR trial OR experiment OR “control group” OR “controlled trial” OR "quasi‐experiment” OR random*)	June 13, 2017
Database of Research on International Education	("trauma‐informed" OR "trauma‐sensitive" OR “trauma services” OR trauma OR PTSD OR “post‐traumatic stress disorder”) AND (“elementary school” OR “primary school” OR “high school” OR “secondary school” OR “middle school” OR kindergarten OR "pre‐kindergarten" OR child* OR youth OR adolescent OR school) AND (evaluation OR intervention OR treatment OR outcome OR program OR trial OR experiment OR “control group” OR “controlled trial” OR "quasi‐experiment” OR random*)	June 13, 2017
Education Source	KW ("trauma‐informed" OR "trauma‐sensitive" OR “trauma services” OR trauma OR PTSD OR “post‐traumatic stress disorder”) AND (“elementary school” OR “primary school” OR “high school” OR “secondary school” OR “middle school” OR kindergarten OR "pre‐kindergarten" OR child* OR youth OR adolescent OR school) AND (evaluation OR intervention OR treatment OR outcome OR program OR trial OR experiment OR “control group” OR “controlled trial” OR "quasi‐experiment” OR random*)	June 13, 2017
ERIC	SU ("trauma‐informed" OR "trauma‐sensitive" OR “trauma services” OR trauma OR PTSD OR “post‐traumatic stress disorder”) AND (“elementary school” OR “primary school” OR “high school” OR “secondary school” OR “middle school” OR kindergarten OR "pre‐kindergarten" OR child* OR youth OR adolescent OR school) AND (evaluation OR intervention OR treatment OR outcome OR program OR trial OR experiment OR “control group” OR “controlled trial” OR "quasi‐experiment” OR random*)	June 13, 2017
MEDLINE	((trauma‐informed or trauma‐sensitive or trauma services or trauma or PTSD or post‐traumatic stress disorder) and (elementary school or primary school or high school or secondary school or middle school or kindergarten or pre‐kindergarten or child* or youth or adolescent or school) and (evaluation or intervention or treatment or outcome or program or trial or experiment or control group or controlled trial or quasi‐experiment or random*)).hw.	June 13, 2017
ProQuest Dissertations and Theses	diskw(("trauma‐informed" OR "trauma‐sensitive" OR "trauma services" OR trauma OR pood OR "post‐traumatic stress disorder") AND ("elementary school" OR "primary school" OR "high school" OR "secondary school" OR "middle school" OR kindergarten OR "pre‐kindergarten" OR child* OR youth OR adolescent OR school) AND (evaluation OR intervention OR treatment OR outcome OR program OR trial OR experiment OR "control group" OR "controlled trial" OR "quasi‐experiment" OR random*))	June 14, 2017
PsycINFO	((trauma‐informed or trauma‐sensitive or trauma services or trauma or PTSD or post‐traumatic stress disorder) and (elementary school or primary school or high school or secondary school or middle school or kindergarten or pre‐kindergarten or child* or youth or adolescent or school) and (evaluation or intervention or treatment or outcome or program or trial or experiment or control group or controlled trial or quasi‐experiment or random*)).sh	June 14, 2017
Social Science Citation Index	("trauma‐informed" OR "trauma‐sensitive" OR “trauma services” OR trauma OR PTSD OR “post‐traumatic stress disorder”) AND (“elementary school” OR “primary school” OR “high school” OR “secondary school” OR “middle school” OR kindergarten OR "pre‐kindergarten" OR child* OR youth OR adolescent OR school) AND (evaluation OR intervention OR treatment OR outcome OR program OR trial OR experiment OR “control group” OR “controlled trial” OR "quasi‐experiment” OR random*)	June 14, 2017
CINAHL	SU ("trauma‐informed" OR "trauma‐sensitive" OR “trauma services” OR trauma OR PTSD OR “post‐traumatic stress disorder”) AND (“elementary school” OR “primary school” OR “high school” OR “secondary school” OR “middle school” OR kindergarten OR "pre‐kindergarten" OR child* OR youth OR adolescent OR school) AND (evaluation OR intervention OR treatment OR outcome OR program OR trial OR experiment OR “control group” OR “controlled trial” OR "quasi‐experiment” OR random*)	June 14, 2017

1) Intervention/condition: Trauma‐informed OR trauma‐sensitive OR “trauma services” OR trauma OR PTSD OR “post‐traumatic stress disorder”
  AND
2) Targeted population: “elementary school” OR “primary school” OR “high school” OR “secondary school” OR “middle school” OR kindergarten OR pre‐kindergarten OR child* OR youth OR adolescent OR school
3) Report type: Evaluation OR intervention OR treatment OR outcome OR program OR trial OR experiment OR “control group” OR “controlled trial” OR quasi‐experiment” OR random*

#### 5.2.3 Research registers and websites

a. Cochrane Collaboration Library
b. Database of Abstracts of Reviews of Effectiveness
c. National Technical Information Service
d. System for Information on Gray Literature
e. Evidence for Policy Practice Information and Coordinating Center (EPPI‐Center)

#### 5.2.4 Gray literature sources

a. Social Science Research Network
b. Conference abstracts and proceedings will be reviewed to identify potentially relevant studies. Conference searches will include:
  i. The Society for Research on Educational Effectiveness (https://www.sree.org/pages/conferences/index.php)
  ii. American Educational Research Association Repository (http://www.aera.net/EventsMeetings/tabid/10063/Default.aspx.)
  iii. Society for Research on Child Development (SCRD)
  iv. Society for Research on Adolescence (SRA)
  v. International Society for Traumatic Stress Studies (https://www.istss.org/)
  vi. Tampa Children's Mental Health Research and Policy Conference
  vii. School Social Work Association of America (National School Social Work Conference)

#### 5.2.5 Clearinghouses, research centers and disciplinary and government websites

a. The US Department of Education's web site contains reports of funded programs and initiatives: http://www2.ed.gov/about/offices/list/opepd/ppss/reports.html
b. The Institution of Education Sciences, What Works Clearinghouse contains reports of intervention investigations: http://ies.ed.gov/funding/grantsearch/index.asp
c. Trauma and Learning Policy Initiative: traumasensitiveschools.org
d. National Child Traumatic Stress Network: www.nctsn.org
e. American Public Health Association
f. Association for Psychological Science
g. American Psychological Association
h. International Society for Traumatic Stress Studies

#### 5.2.6 Reference lists and contact with authors

The reference lists from prior reviews and studies retrieved for full‐text screening were reviewed for potential studies. We had planned to conduct forward citation searches for all included studies; however, no studies met inclusion criteria. We also emailed (or attempted to email) first/corresponding authors of all full‐text reports screened for inclusion.

### 5.3 Data collection and analysis

#### 5.3.1 Selection of studies

One reviewer conducted the initial search in all sources and examined titles and abstracts. Searches in electronic databases were conducted June 13–14, 2017. Searches in gray literature sources, conference abstracts and proceedings and other websites were completed by June, 2017. Review of bibliographies of all full‐text screened reports was completed on September 14, 2017 and authors of all full‐text screened reports were contacted via email September 18, 2017. Titles and abstracts of reports that were obviously ineligible (nonempirical report, book review, editorial, adult participants, prior to 2006, etc.) were discarded. For those that were not obviously ineligible, the reviewer uploaded the full citation and the full‐text report into Covidence (2016). Two reviewers then independently screened each of the full‐text reports for eligibility using a screening instrument (see review protocol, Maynard et al., 2017). Covidence identified disagreements which the two reviewers then resolved through discussion and consensus.

#### 5.3.2 Data extraction and management

No studies met inclusion criteria, thus data was not extracted from studies.

#### 5.3.3 Assessment of risk of bias in included studies

No studies met inclusion criteria, thus risk of bias was not assessed.

#### 5.3.4 Measures of treatment effect

No studies met inclusion criteria, thus no effect sizes were calculated.

## 6 RESULTS

### 6.1 Description of studies

#### 6.1.1 Results of the search

**Figure 2:**
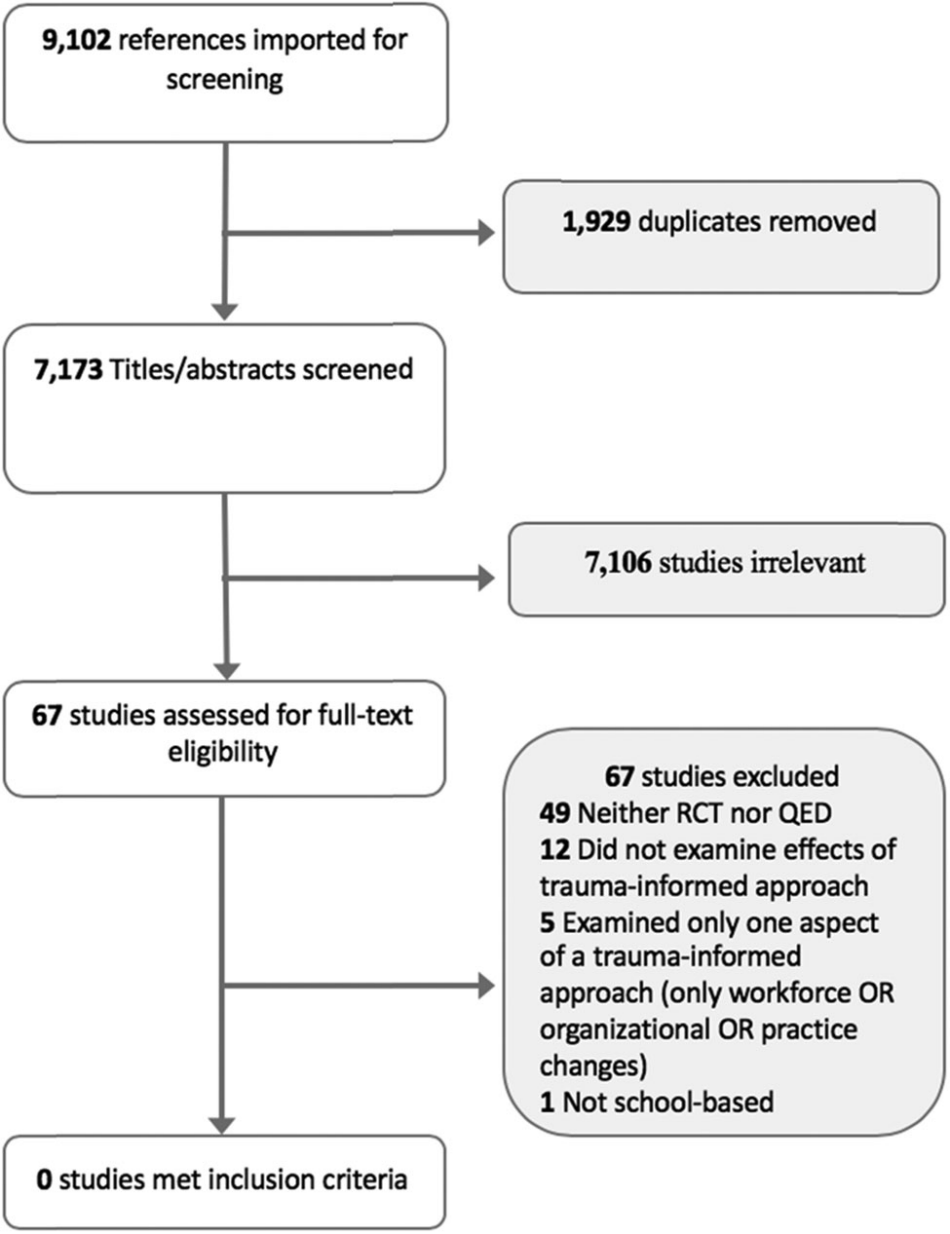
Search and selection flowchart

A total of 9,102 references from all searches were imported to Covidence for title and abstract screening. After removal of 1,929 duplicates, 7,173 titles/abstracts were screened by one reviewer and 7,106 reports were excluded. Two reviewers then screened the full text of the remaining 67 reports. All 67 reports were excluded: 49 were neither an RCT nor QED; 12 did not examine effects of a trauma‐informed approach; 5 examined only one aspect of a trauma‐informed approach (only workforce OR organizational OR practice changes); one was not a school‐based intervention. Some studies may have been excluded for multiple reasons; however, only the first (primary) reason for exclusion was recorded. See Figure 2 for flowchart of the search and selection process. A full list of reports excluded at the full‐text screening stage can be found in References to Excluded Studies in the References section.

No studies met criteria for inclusion in this review.

### 6.2 Risk of bias in included studies

No studies met inclusion criteria, thus no studies were assessed for risk of bias.

### 6.3 Synthesis of results

No studies met inclusion criteria for this review, thus no synthesis was conducted.

## 7 DISCUSSION

### 7.1 Summary of main results

Trauma‐informed approaches are being promoted and used across child‐serving systems, and the number of states and school districts adopting trauma‐informed approaches in schools in the US and other countries is growing rapidly (Overstreet & Chafouleas, 2016). While the premise of a trauma‐informed schools approach is a noble one, it is unclear as to whether the promise of this framework is actually delivering the types of systemic and programmatic changes intended and if those changes are resulting in the outcomes the proponents of a trauma‐informed approach in schools hoped for. The purpose of this systematic review was to find, describe, evaluate and synthesize effects of trauma‐informed approaches in schools to inform policy and practice. Despite our extensive search for studies, we found no studies that met criteria for inclusion in this review. While there are a number of publications that describe trauma‐informed approaches, advocate for the need for trauma‐informed approaches, and discuss the potential benefits of adopting such an approach in schools, there have been no rigorous evaluations of trauma‐informed approaches in schools that we could find.

While the paucity of rigorous research in this area is disappointing, it is not altogether surprising. The adoption of a trauma‐informed approach is relatively new and it is likely that there has not been sufficient time for the research to catch up to the enthusiasm for this approach in schools. Furthermore, conducting rigorous research on multi‐component and multi‐tiered approaches can be complex and expensive, often requiring large grants to help fund the research, which can also delay the conduct of rigorous research.

### 7.2 Overall completeness, applicability, and quality of evidence

We found no studies that met eligibility criteria, thus the evidence in this area is completely lacking.

### 7.3 Limitations and potential biases in the review process

While this review sheds light onto the lack of evidence available to inform policy and practice regarding trauma‐informed approaches in schools, the present study is not without limitations. Despite our attempts at a comprehensive search process, there is the possibility that we may have not captured every potentially eligible study; however, we believe the risk is quite small given the extensive search process. Anecdotally, we are aware of some ongoing studies, but there are no protocols published to provide more information about these studies in this review. This review was limited to studies that included at least two of the three components of a trauma‐informed approach (workforce/PD, organization change or practice change), thus we may have excluded studies that authors themselves or others may consider a trauma‐informed approach.

## 8 AUTHORS’ CONCLUSIONS

This empty review comes at an admittedly early stage in American schools' embrace of the trauma‐informed approach. Many innovations in education start with a great deal of excitement and moral fervor that is often not matched by rigorous evaluation of the interventions or curriculum being implemented (Walker, 2004). The trauma‐informed approach appears to be no exception; despite the increasing adoption of trauma‐informed approaches in schools, we found no rigorous evaluations of trauma‐informed approaches in schools that might indicate whether or how this approach works to address the various impacts of trauma on young people, families, and educators. This review also could not provide any strong evidence to date of what the school‐level impacts are (if any) of implementing this approach, such as improved academic and behavioral outcomes and reduced teacher burnout, raising concern about the possibility that the trauma‐informed “movement” might collapse or fizzle without any solid evidence to support its goals, as so many other well‐intentioned school mental health interventions have in the past (Kelly, Raines, Stone & Frey, 2010).

While we have noted in our review that there are individual programs with evidence to support their effectiveness as school‐based interventions for students dealing with trauma, there appears to be an implication that those programs alone “count” as evidence that using a trauma‐informed approach itself works in K‐12 education. We have observed this in our own home areas, and in several of the excluded studies that claimed to be using an evidence‐informed approach, but were evaluating a specific program, like CBITS, SPARCS, and Bounce Back. These targeted prevention or intervention programs may be effective in reducing trauma symptoms, but do not constitute a trauma‐informed approach as defined by SAMHSA. It is important that a clear definition of what constitutes a trauma‐informed approach in schools be established and that schools, and evaluators, be clear in discerning between whether they are truly implementing a trauma‐informed approach, or implementing an evidence‐informed intervention to prevent or treat trauma.

This review also points to the persistent problem of scale and diffusion of innovative practices in education and sheds some possible light on how this is playing out with the trauma‐informed approach movement. It is unfortunately not uncommon for education innovations to be embraced and adopted on a relatively large scale prior to rigorous evidence demonstrating positive effects. Indeed, this is what happened with PBIS/RTI/MTSS. The public health prevention framework that first emerged in the early 1990s was codified into federal law in the late 1990s and early 2000s, and then over time slowly developed a strong evidence base to support their effectiveness in addressing academic, behavioral, and emotional problems for youth in K‐12 schools (Bradshaw, Koth, Thornton, & Leaf, 2009; Bradshaw, Koth, Bevans, Ialongo, & Leaf, 2008; Horner & Sugai, 2015). Today it is estimated that the three‐tier frameworks of PBIS/RTI/MTSS have been implemented in over 19,000 American schools, making them one of the most scaled‐up educational interventions in American schools (Barrett, Eber, & Weist, 2013). The trauma‐informed approach may very well follow this same trajectory, but we encourage greater attention to promoting rigorous evaluation of trauma‐informed approaches in schools sooner rather than later.

In considering the issue of scaling up an innovative strategy like the trauma‐informed approach, we can draw from Clark & Dede's scaling framework for educational innovations (2009). However, of the five components of Clark & Dede's scaling framework for educational innovations, which includes depth/effectiveness of the innovation followed by the innovation's sustainability, spread, adoption, and evolution/further adaptation, the only one that appears to be active with the trauma‐informed approach is the “spread” of the trauma‐informed framework; however, loosely it appears to be presently defined. In just a short period of time, the trauma‐informed approach has already begun to “spread” into American K‐12 education at a rapid clip. This rapid spread has the potential to quickly become another example of an education trend that falters without evidence to sustain them (Baker, 2007; Dearing et al., 2015). This empty review demonstrates that the other components of Clark & Dede's scaling framework, largely effectiveness of the innovation, are absent from current literature on trauma‐informed approaches in schools.

### 8.1 Implications for practice and policy

From this review, it seems like the most prudent thing for school leaders, policymakers, and school mental health professionals to do would be proceed with caution in their embrace of a trauma‐informed approach as an overarching framework and conduct rigorous evaluation of this approach. We simply do not have the evidence (yet) to know if this works, and indeed, we do not know if using a trauma‐informed approach could actually have unintended negative consequences for traumatized youth and school communities. We also do not have evidence of other potential costs in implementing this approach in schools, whether they be financial, academic, or other opportunity costs, and whether benefits outweigh the costs of implementing and maintaining this approach in schools. That said, calling for caution in adopting TIC in schools does not preclude schools from continuing to implement evidence‐informed programs that target trauma symptoms in youth, or that they should simply wait for the research to provide unequivocal answers. The benefit of the trauma‐informed approach being made freely available by SAMHSA and other policymakers is that these components can form the basis for a school (or school district) to begin to adapt and apply this approach in schools.

An additional potential space for implementing a trauma‐informed approach could be within the various 3‐tier models currently active in schools (often referred to as multi‐tiered systems of supports [MTSS]) to give some form and structure to these efforts. Indeed, recent scholarship has argued for the trauma‐informed approach to be embedded within MTSS to take advantage of the primary prevention focus inherent in MTSS Tier 1 and Tier 2 efforts, along with the use of data via screening tools to identify students who are impacted by trauma (Cavanaugh, 2016; Stephan, Suagi, Lever, & Connors, 2015; Zakzeski, Ventresco, & Jaffe, 2017). The process of screening students for trauma is not without its own controversy; however, as parent groups and school stakeholders sometimes oppose the idea of screening youth in schools for issues that they believe are the domain of parents and mental health systems to handle (Dowdy, Ritchey, & Kamphaus, 2010).

### 8.2 Implications for research

The implications for research are clear: Trauma‐informed interventions need to be rigorously evaluated. Anecdotally, we are aware of some studies currently underway that are trying to evaluate various components of the trauma‐informed approach in schools; however, neither protocols nor the completed studies have been published. Given the complexity of this approach and knowledge about research of other multi‐component and MTSS in schools, such as Positive Behavioral Interventions and Supports and Response to Intervention, researchers could draw from lessons learned in the conduct of research with these approaches to help inform future studies of trauma‐informed approaches. We also encourage studies that examine the implementation of trauma‐informed approaches in schools. Examining what schools are doing, how they are implementing trauma‐informed approaches, and variations in components being included is important to understanding whether and how trauma‐informed school approaches work and what is required to successfully implement this approach in schools.

### 8.3 Roles and responsibilities

Please give brief description of content and methodological expertize within the review team. The recommended optimal review team composition includes at least one person on the review team who has content expertize, at least one person who has methodological expertize and at least one person who has statistical expertize. It is also recommended to have one person with information retrieval expertize.

Who is responsible for the below areas? Please list their names:

- Content: All authors were responsible for the substantive content related to trauma‐informed schools.
- Systematic review methods: Maynard has significant experience and expertize in systematic review methods. Farina, Dell, and Kelly have had training in and experience conducting systematic reviews.
- Statistical analysis: Maynard has been trained in meta‐analytic techniques and has conducted several meta‐analyses.
- Information retrieval: All authors are experienced in information retrieval. Maynard and Farina consulted with the information retrieval specialist at Saint Louis University in the planning and execution of the search strategy. Farina and Dell executed the search and selection procedures for this review.

## PLANS FOR UPDATING THE REVIEW

Maynard will be responsible for developing a plan for updating the review in approximately 3 years.

## SOURCES OF SUPPORT

The review team received funding from the C2 ECG through a mini‐grant to support the conduct of this review.

## CONFLICT OF INTERESTS

The authors declare that there are no conflict of interests.
